# Greener Choices in Biliary Imaging: A Prospective Carbon Footprint Comparison of Endoscopic Ultrasound Versus Magnetic Resonance Cholangiopancreatography for Intermediate-Likelihood Choledocholithiasis

**DOI:** 10.14309/ctg.0000000000001098

**Published:** 2026-09-08

**Authors:** Hardik Rughwani, Nitin Jagtap, David M. de Jong, Abhishek Tyagi, Tharani Putta, Ashirwad Pasumarthy, Abul Khair Khan, Rakesh Kalapala, Mohan Ramchandani, Sundeep Lakhtakia, D. Nageshwar Reddy

**Affiliations:** 1 Department of Gastroenterology, AIG Hospitals, Hyderabad, India;; 2 Department of Gastroenterology and Hepatology, Erasmus MC, University Medical Center, Rotterdam, the Netherlands;; 3 Department of Artificial Intelligence and Computer Vision, AIG Hospitals, Hyderabad, India;; 4 Department of Radiology, AIG Hospitals, Hyderabad, India.

**Keywords:** carbon footprint, EUS, MRCP, choledocholithiasis, green endoscopy, sustainability

## Abstract

**INTRODUCTION:**

Health care accounts for nearly 5% of global greenhouse gas emissions, and medical imaging is among its most energy-intensive activities. For intermediate-likelihood common bile duct stones, endoscopic ultrasound (EUS) and magnetic resonance cholangiopancreatography (MRCP) are both guideline-endorsed with comparable diagnostic accuracy, yet their environmental costs have never been directly compared.

**METHODS:**

In this prospective study, we measured the per-procedure carbon footprint of EUS and MRCP using the Greenhouse Gas Protocol and India's grid emission factor. We recorded EUS procedure times individually; MRCP emissions were derived from a validated power model that captures the continuous cryocooler load maintaining magnet superconductivity around the clock, with shared infrastructure allocated by real case volume. We performed sensitivity analyses across scanner utilization, energy-allocation method, scan duration, and grid carbon intensity. The study followed the European Society of Gastrointestinal Endoscopy Endoscopic Sustainability PrimAry Reporting Essentials reporting standard.

**RESULTS:**

EUS generated 4.53 kg CO_2_e per procedure and MRCP 18.90 kg CO_2_e per procedure, making MRCP 4.2 times more carbon-intensive. The continuously running cryocooler was the single largest fixed contributor to MRCP emissions (23.4% in standby alone), a 24/7 load absent in EUS. Choosing EUS for 108 patients saved 1,552 kg CO_2_e, equivalent to the annual carbon uptake of 74 mature trees. EUS remained the lower-carbon option across sensitivity analyses, although the advantage narrowed and could reverse on low-carbon grids.

**DISCUSSION:**

For bile duct stone evaluation, EUS offers the same diagnostic answer at substantially lower carbon cost where grid electricity is carbon-intensive, adding environmental impact to accuracy, safety, and cost in the decision.

## Graphical abstract

**Figure:**
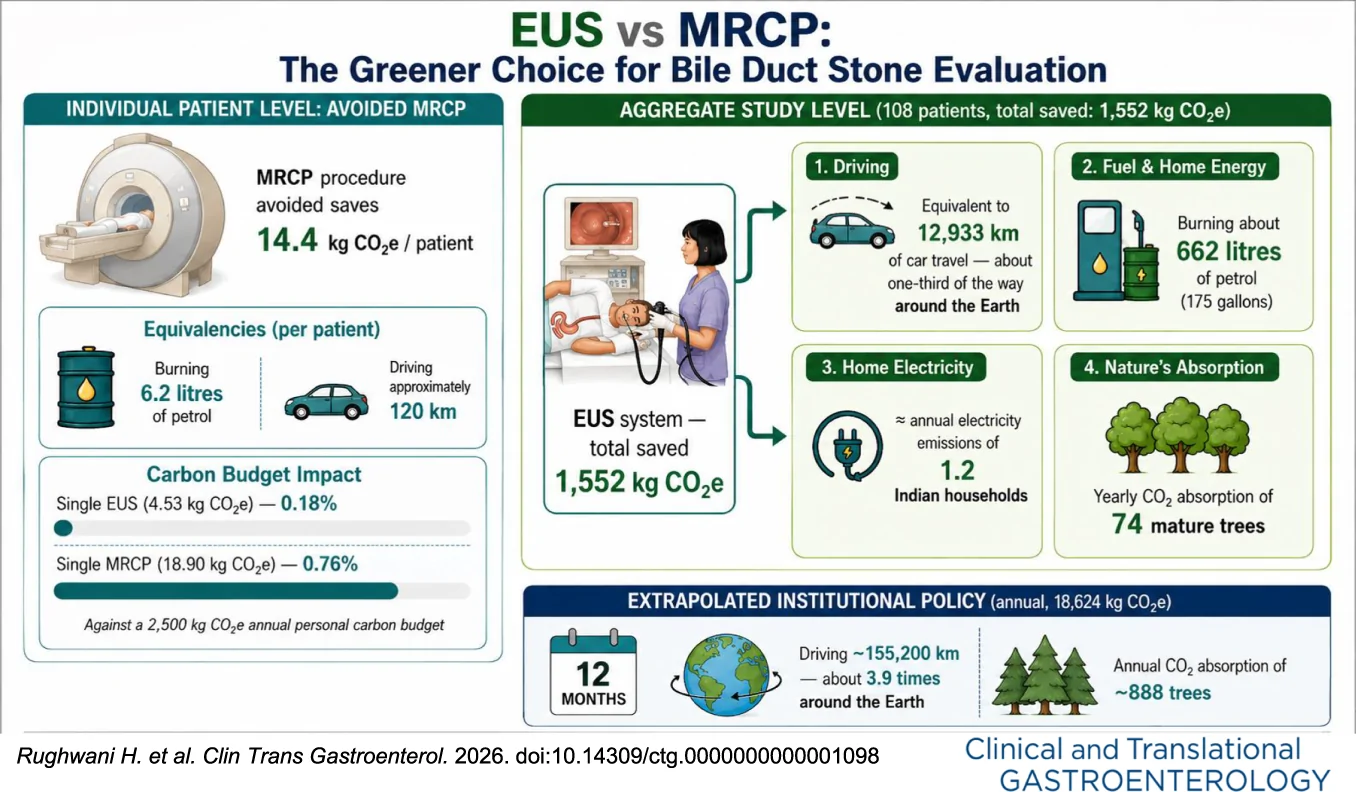

## INTRODUCTION

Healthcare systems worldwide account for approximately 5% of global greenhouse gas (GHG) emissions, and medical imaging is among the most energy-intensive areas of clinical care (1,2). Within the field of gastroenterology, the push for greener practice has gathered pace, particularly after the European Society of Gastrointestinal Endoscopy (ESGE) and the European Society of Gastroenterology and Endoscopy Nurses and Associates released their joint position statement on reducing the environmental footprint of gastrointestinal (GI) endoscopy (3). Building on this momentum, the first detailed carbon footprint assessments of GI endoscopy from France (4), the USA (5), and India (6) were published, pinpointing electricity, water, and waste as the main sources of emissions in a tertiary center.

Awareness, however, has outpaced action: in the LEAFGREEN survey, 86% of endoscopy professionals accepted climate change as real and anthropogenic, yet only 4% of units had ever estimated their own carbon footprint (7).

Choledocholithiasis, the presence of stones in the common bile duct (CBD), affects a significant proportion of patients with gallstones and requires timely, accurate diagnosis. Current ESGE guidelines endorse either endoscopic ultrasound (EUS) or magnetic resonance cholangiopancreatography (MRCP) for patients in the intermediate-likelihood category (8). A landmark randomized controlled trial by Jagtap et al (9) showed that the 2 modalities performed comparably: EUS achieved a sensitivity of 95.7% and specificity of 98.5%, whereas MRCP achieved 92.3% and 95.7%, respectively. Because the diagnostic yields are broadly similar (EUS was marginally more sensitive in that trial), the choice between them often comes down to availability, cost, and patient preference. Unfortunately, environmental impact has never been part of that conversation.

Several important differences exist between EUS and MRCP. EUS relies on compact, low-power ultrasound transducers housed in a standard endoscopy room. MRCP, by contrast, uses a 3.0 T superconducting magnetic resonance imaging (MRI) scanner, one of the most electricity-demanding machines in routine clinical practice. The MRI magnet cannot simply be turned off; it must be held at approximately 4.2 K (Kelvin; approximately −269 °C) around the clock by a cryocooler compressor drawing 9 kW continuously, 24 hours a day, regardless of scan volume. Therefore, this study aimed to conduct a prospective, patient-level comparison of the carbon footprints of EUS and MRCP, incorporating all relevant components that previous studies had not examined.

## METHODS

### Study design and setting

This prospective observational study was conducted at the Asian Institute of Gastroenterology (AIG Hospitals), Hyderabad, India, during January 2026. The study was registered with ClinicalTrials.gov (NCT07283185) and approved by the Institutional Ethics Committee, Asian Institute of Gastroenterology (IEC-AIG; Approval Number: AIG/IEC-BH&R 74/09.2025-1). We obtained written informed consent from every patient before enrolment. AIG Hospitals is a high-volume quaternary gastroenterology center. Over the study month, 486 EUS procedures were performed across 27 working days in 2 dedicated EUS rooms, and 676 abdominal MRI/MRCP scans were performed over all 31 calendar days on 1 machine operating around the clock.

### Clinical protocol and patient allocation

The allocation between EUS and MRCP was not randomized. Patients with suspected intermediate-likelihood choledocholithiasis (as defined by the ESGE criteria) were evaluated by the attending endoscopist, and the choice between EUS and MRCP was made at the physician's discretion based on (i) the clinical presentation, (ii) availability of prior imaging studies or laboratory reports at the time of consultation, (iii) probability of progressing directly to therapeutic endoscopic retrograde cholangiopancreatography (ERCP) in the same session, and (iv) institutional scheduling. In our institutional pathway, patients with a higher clinical suspicion and those presenting during working hours typically underwent EUS, which allowed same-session ERCP if stones were confirmed. MRCP was preferred when cross-sectional anatomy was required for surgical planning or when EUS scheduling was not feasible. All subsequent interventions (ERCP with stone extraction, cholecystectomy, or conservative management) were performed in accordance with standard institutional guidelines, regardless of the diagnostic modality used.

### Emission factors for EUS

Emissions for each EUS procedure were calculated using the GHG Protocol methodology, following the same framework we used in our earlier institutional carbon footprint assessment. We considered 3 categories of emissions: electricity, water, and waste.

The electricity component was the most detailed. We obtained power ratings for every device in the EUS suite directly from the hospital's Engineering and Maintenance department and cross-checked them against manufacturer specifications. These included the endoscope system (Olympus UCT-180 echoendoscope; 1.6 kW), the room light source (0.5 kW, running 18 hours per day), the procedure monitor (1.5 kW, 10 hours per day), the room air conditioning (1.0 kW, 18 hours per day), and the workstation (1.0 kW, 10 hours per day). The reprocessing area added additional load from the endoscope wash machine (4.5 kW), the reprocessing room light source (0.5 kW), and the air compressor used for scope drying (2.0 kW). Finally, the bedsheet wash machine contributed a small amount (20 kWh per month). Crucially, active endoscope scanning time was not estimated but recorded individually for every case, logged as in-time and out-time during the procedure, so machine-on emissions reflected the true duration of each examination rather than a fixed assumption.

The water component captured the reverse-osmosis water consumed during scope reprocessing (75 L per wash cycle), together with the water used to launder each patient's bedsheet (12 L per patient). These were treated as Scope 3 emissions.

Finally, the waste and consumables component included single-use devices, biopsy containers, and disposable accessories used during the procedure, with emissions computed using the Department for Energy Security and Net Zero 2023 emission factors (10).

Sedation-related materials were also captured. Across the cohort, 180 propofol glass vials (22 g each; 3.96 kg of glass in total) were used and included as a consumable, applying a container-glass life-cycle emission factor (∼0.85 kg CO_2_e per kg); this added approximately 0.031 kg CO_2_e per procedure (<1% of the EUS footprint). Intravenous consumables, oxygen, monitoring, and recovery-room utilization are captured within the waste, water, and shared-infrastructure components described above. The manufacturing footprint of the propofol drug substance itself was not separately modeled and is expected to be minor relative to the intermodality difference.

### Emission factors for MRCP

MRI/magnetic resonance enterography/MRCP calculations used the Philips Ingenia Elition 3.0 T scanner, which operates in 3 distinct power states: 20 kW average during active scanning (gradient coils, radiofrequency transmitter, reconstruction), 13 kW in ready-to-scan mode (patient on table, intersequence planning), and 9 kW in standby (cryocooler compressor maintaining the superconducting magnet at 4.2 K, running continuously). MRI scan duration ranged from 20 to 40 minutes, depending on protocol; an average 30-minute appointment comprising 20 minutes of active scanning and 10 minutes of ready-to-scan overhead was used. For 676 scans over 31 days (∼22 scans/d), daily scanner energy was: 20 kW × 7.27 hours active scanning (145.4 kWh) + 13 kW × 3.63 hours ready-to-scan (47.2 kWh) + 9 kW × 13.10 hours standby (117.9 kWh) = 310.5 kWh/d. Room infrastructure (AC 2 kW, lighting 1 kW, monitor and printer 5 kW, all 24 hr/d) was accounted for separately at 192 kWh/d. Per-scan allocation: scanner 14.24 kWh + room 8.80 kWh = 23.04 kWh total. Waste and consumables were included using Department for Energy Security and Net Zero 2023 emission factors. The three-state power model is consistent with Heye et al (11), Woolen et al (12), and Chodorowski et al (13), the last of whom reported that 72%–91% of MRI energy consumption is nonproductive. For both modalities, we applied the India national grid emission factor of 0.82 kg CO_2_e per kWh (Central Electricity Authority, User Guide Version 19, 2023–24) (14) to all electricity inputs, and used a water emission factor of 10.6 kg CO_2_e per kiloliter for all water consumption. Per-scan emissions were apportioned across the scanner's real total workload of 676 MRI examinations (MRCP, MR enterography, and contrast-enhanced abdominal MRI), the appropriate basis for allocating the scanner's fixed 24/7 loads; a dedicated MRCP sequence is typically shorter than this average and would sit toward the lower end of the 20–40-minute range.

### Outcomes

The primary outcome was the per-procedure carbon footprint (kg CO_2_e) of EUS compared with MRCP. Secondary outcomes were (i) the component-level breakdown of emissions for each modality, (ii) total annualized savings if EUS were adopted preferentially over MRCP for all eligible intermediate-likelihood choledocholithiasis patients, and (iii) the environmental equivalents of the observed savings in everyday terms.

### Statistical analysis and data collection

This study was designed, conducted, and reported in accordance with the ESGE Endoscopic Sustainability PrimAry Reporting Essentials checklist (15), the recommended reporting standard for GI endoscopy sustainability studies. Two trained research associates collected procedure-level data prospectively on a structured case report form and cross-checked them against the hospital's electronic medical record system. Continuous variables are presented as mean (SD) and range. Categorical variables are presented as counts and percentages. Per-procedure emissions for each modality are reported with a component-wise breakdown. Data were analyzed using Microsoft Excel (version 16.0; Microsoft Corporation, Redmond, WA) in accordance with the GHG Protocol framework.

### Sensitivity analysis

Because some MRCP inputs were taken from equipment specifications rather than measured for each patient, we checked how much the comparison depended on 4 things: how busy the scanner is (5–40 scans per day), how the scanner's constant standby energy is counted (spread across every scan, or counted only as the extra energy each scan adds), how long the scan takes (20–40 minutes), and how clean the local electricity grid is (from India's 0.82 down to low-carbon grids at 0.03–0.05 kg CO_2_e/kWh). For the throughput scenarios, we modeled per-scan MRCP emissions as the energy each scan adds plus the scanner's fixed daily standby and room load divided by the number of scans per day, so that these constant loads are shared across whatever volume a center runs. Each scenario is given as a single estimate rather than a confidence interval because the main inputs are fixed equipment power ratings rather than sampled measurements.

## RESULTS

### Study population

During January 2026, 108 patients underwent EUS for suspected intermediate-likelihood choledocholithiasis. The mean EUS procedure duration, recorded individually as in-time and out-time, was 13.5 minutes (SD 5.9, range 1–28 minutes), with a total active endoscope usage of 25 hours across the cohort. During the same calendar month, 676 MRI examinations of the upper abdomen (including MRI abdomen with contrast, MR enterography, and MRCP) were performed on the Philips Ingenia Elition 3.0 T scanner, averaging 22 scans per day over 31 days (16). Table 1 summarizes study characteristics.

**Table 1.** Study characteristics

Parameter	EUS	MRCP
Study period	January 2026 (27 working days)	January 2026 (31 calendar days)
Total procedures	486	676
CBD stone cases analyzed	108	N/A (per-scan allocation)
Daily procedure load	18.0 per day (2 rooms)	21.8 per day (1 machine, 24/7)
Machine	Olympus UCT-180 linear EUS	Philips Ingenia Elition 3.0 T MRI
Mean procedure duration	13.5 min (SD 5.9, range 1–28)	30 min (20 active + 10 ready)
Operating hours	10 hr/d, Mon–Sat	24 hr/d, 7 d/wk
Sedation	Deep sedation (propofol)	Not required
Scope reprocessing	75 L water/cycle, 4.5 kW wash	Not applicable

CBD, common bile duct; EUS, endoscopic ultrasound; MRCP, magnetic resonance cholangiopancreatography; MRI, magnetic resonance imaging.

### Per-procedure carbon footprint

Each EUS procedure generated 4.53 kg CO_2_e. Each MRCP generated 18.90 kg CO_2_e and consumed 23.04 kWh. MRCP therefore produced approximately 4.2 times the per-procedure greenhouse-gas emissions of EUS (Figure 1).

**Figure 1.**
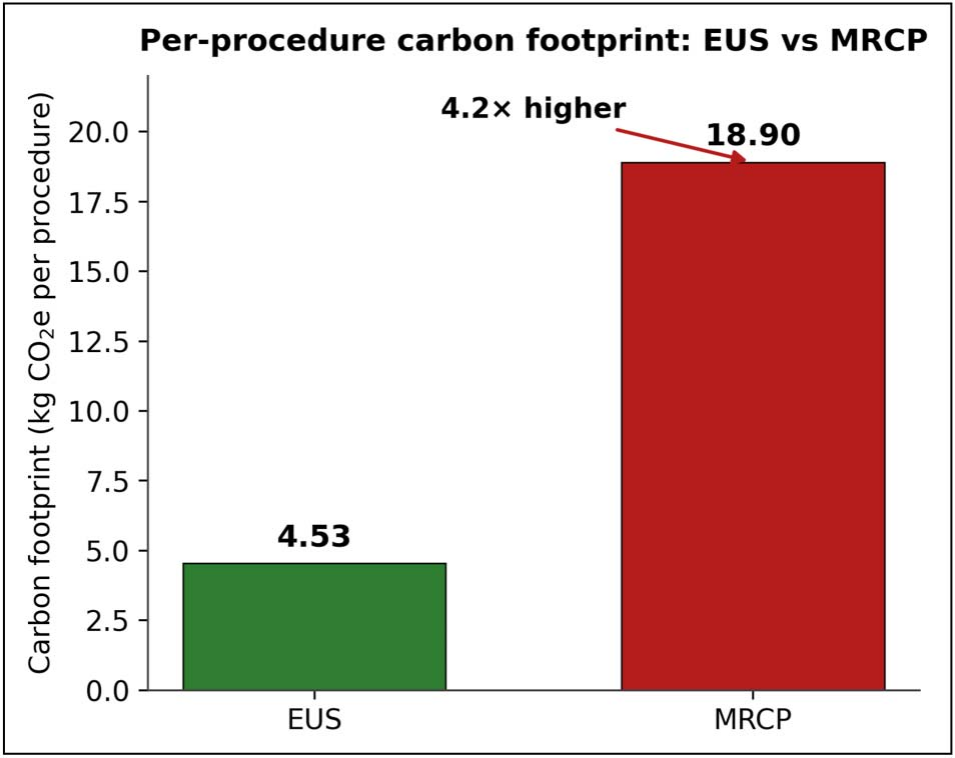
Per-procedure carbon footprint: EUS vs MRCP. MRCP generates 4.2 times the emissions of EUS (18.90 vs 4.53 kg CO_2_e per procedure) for the equivalent diagnostic indication. CO_2_e, carbon dioxide equivalent; EUS, endoscopic ultrasound; MRCP, magnetic resonance cholangiopancreatography.

#### EUS emission breakdown.

For the EUS pathway, shared room infrastructure (room air conditioning, lighting, workstation, and monitor) contributed the largest share (52.2%; 2.369 kg CO_2_e). Scope reprocessing was the next largest contributor at 37.3% (1.691 kg CO_2_e), with its electricity and water components shown separately: reprocessing electricity (wash machine, reprocessing-room light, and air compressor) at 19.8% (0.896 kg CO_2_e, Scope 2) and scope-wash water at 17.5% (0.795 kg CO_2_e, Scope 3). The active endoscope scan itself contributed only 6.7% (0.304 kg CO_2_e), reflecting the low 1.6 kW draw of the Olympus endoscope system and the short mean procedure duration. Bedsheet laundering added a further 0.161 kg CO_2_e (3.6%; electricity plus Scope 3 water), and waste and consumables added 0.010 kg CO_2_e per procedure (Table 2, Figure 2).

**Table 2.** EUS emissions breakdown per procedure (4.53 kg CO_2_e total)

Component	kg CO_2_e	%
Scope 2: Electricity emissions		
Endoscope system (1.6 kW, active scan)	0.304	6.7
Room light source (0.5 kW, 18 hr/d)	0.410	9.1
Monitor (1.5 kW, 10 hr/d)	0.683	15.1
Room air conditioning (1.0 kW, 18 hr/d)	0.820	18.1
Workstation/computer (1.0 kW, 10 hr/d)	0.456	10.1
Endoscope wash machine (4.5 kW)	0.577	12.7
Reprocessing-room light source (0.5 kW)	0.228	5.0
Air compressor for scope drying (2.0 kW)	0.091	2.0
Bedsheet wash machine (20 kWh/mo)	0.034	0.7
**Subtotal — electricity**	**3.603**	**79.5**
Scope 3: Water emissions (EF 10.6 kg CO_2_e/kL)		
Scope wash water (75 L × 108 cases = 8,100 L)	0.795	17.5
Bedsheet wash water (12 L × 108 = 1,296 L)	0.127	2.8
**Subtotal — water (9,396 L = 9.396 kL)**	**0.922**	**20.3**
Waste and consumables	0.010	0.2
**TOTAL per EUS procedure**	**4.53**	**100**

Bold rows denote the Scope 2 and Scope 3 subheadings, and the total carbon footprint per EUS procedure. CO_2_e, carbon dioxide equivalent; EF, emission factor; EUS, endoscopic ultrasound; kL, kiloliter; kW, kilowatt; kWh, kilowatt-hour.

**Figure 2.**
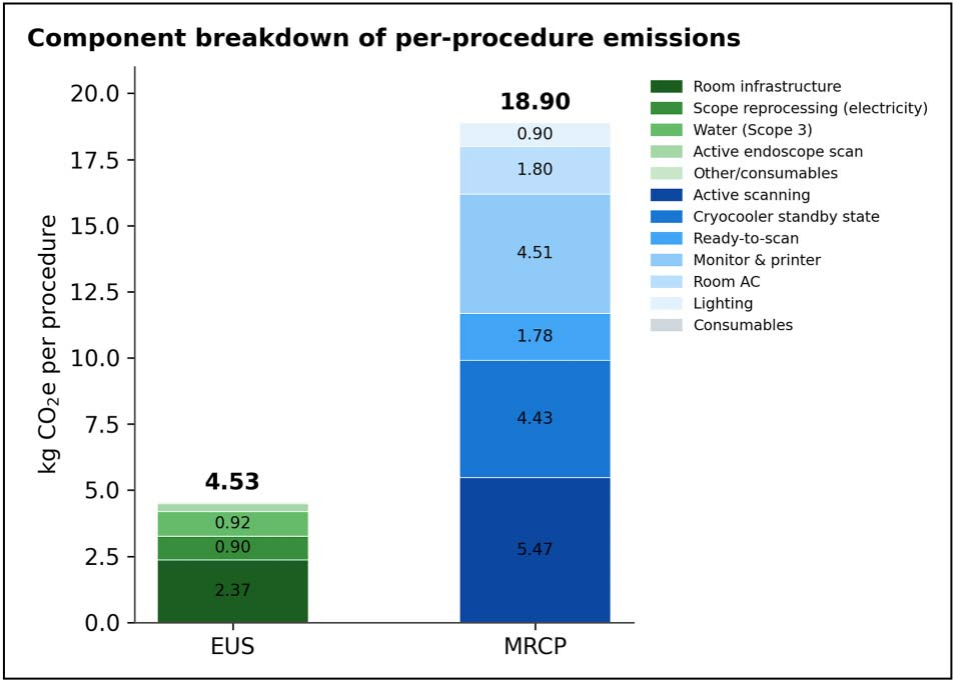
Component breakdown of per-procedure emissions. EUS is dominated by shared room infrastructure (52.2%) and scope reprocessing (37.3%; electricity 19.8% and scope-wash water 17.5%), with the active endoscope scan contributing only 6.7%. MRCP is dominated by the MRI scanner across its 3 power states (active scan 29.0%, cryocooler standby 23.4%, ready-to-scan 9.4%), which together account for 61.7% of emissions; room infrastructure contributes the remaining 38.2%. CO_2_e, carbon dioxide equivalent; EUS, endoscopic ultrasound; MRCP, magnetic resonance cholangiopancreatography.

#### MRCP emission breakdown.

For the MRCP pathway, the MRI scanner, modeled across its 3 power states, accounted for 61.7% of per-scan emissions (11.68 kg CO_2_e). Active scanning was the largest component at 29.0% (5.47 kg CO_2_e), followed by standby power, including the cryocooler, at 23.4% (4.43 kg CO_2_e), and ready-to-scan overhead at 9.4% (1.78 kg CO_2_e). Room infrastructure accounted for the remaining 38.2%, distributed among monitor and printer at 23.9% (4.51 kg CO_2_e), room air conditioning at 9.5% (1.80 kg CO_2_e), and lighting at 4.8% (0.90 kg CO_2_e). Waste and consumables contributed 0.010 kg CO_2_e (Table 3, Figure 2).

**Table 3.** MRCP emissions breakdown per procedure (18.90 kg CO_2_e total)

Component	kg CO_2_e	%
MRI scanner (three-state power model)		
Active scanning (20 kW × 20 min/scan)	5.47	29.0
Standby including cryocooler (9 kW, 24/7)	4.43	23.4
Ready-to-scan overhead (13 kW × 10 min/scan)	1.78	9.4
**Subtotal — scanner**	**11.68**	**61.7**
Room infrastructure (allocated per scan)		
Monitor and printer (5 kW, 24/7)	4.51	23.9
Room air conditioning (2 kW, 24/7)	1.80	9.5
Room lighting (1 kW, 24/7)	0.90	4.8
**Subtotal — room**	**7.21**	**38.2**
Waste and consumables	0.010	0.1
**TOTAL per MRCP procedure**	**18.90**	**100**

The 9 kW baseline (dominated by the cryocooler) runs continuously and is therefore also embedded in the active-scanning and ready-to-scan figures; the 23.4% shown is the standby-state slice only. Expressed as a continuous 24/7 load, the cryocooler alone accounts for approximately 42% of per-scan MRCP emissions. Bold rows denote the Scope 2 and Scope 3 subheadings, and the total carbon footprint per MRCP procedure.

CO_2_e, carbon dioxide equivalent; kW, kilowatt; MRCP, magnetic resonance cholangiopancreatography; MRI, magnetic resonance imaging.

### Cumulative emissions, carbon budget, and environmental equivalents

Across the 108 patients with suspected intermediate-likelihood CBD stones who underwent EUS during the study period, total emissions were 489 kg CO_2_e. If these same 108 patients had been evaluated with MRCP, equivalent emissions would have been 2,041 kg CO_2_e, an absolute saving of 1,552 kg CO_2_e attributable to the EUS pathway, or 14.4 kg CO_2_e per patient (Table 4). Extrapolated over 12 months at our institutional case volume, this corresponds to an annual saving of approximately 18,624 kg CO_2_e.

**Table 4.** Comparative emissions summary

Metric	EUS	MRCP
CO_2_e per procedure (kg)	4.53	18.90
Largest single contributor	Room infrastructure (52.2%)	Active scan (29.0%)
Scope reprocessing (electricity)	0.896 kg (19.8%)	Not applicable
Scope-wash water	0.795 kg (17.5%)	
Cryocooler standby (24/7)	Not applicable	4.43 kg (23.4%)
CO_2_e for 108 procedures (kg)	489	2,041
Fold difference (MRCP vs EUS)	—	**4.2× more carbon-intensive**
Saving for 108 cases (EUS vs MRCP)	**1,552 kg CO**_**2**_ **e saved**	—
Annual saving (12 mo)	**18,624 kg CO**_**2**_ **e**	≈ 74 mature trees

Bold text denotes the column headings and the metric labels in the first column; the fold difference (4.2×) is shown in bold to highlight the primary comparison. CO_2_e, carbon dioxide equivalent; EUS, endoscopic ultrasound; MRCP, magnetic resonance cholangiopancreatography.

To place these figures in perspective, an individual's annual carbon budget compatible with limiting global warming to 1.5 °C is roughly 2,000 to 3,000 kg CO_2_e. Based on a midpoint of 2,500 kg CO_2_e per year, a single EUS procedure consumes only 0.18% of 1 person's annual carbon budget, whereas a single MRCP consumes 0.76%. The 14.4 kg CO_2_e saved per patient by choosing EUS amounts to 0.58% of 1 person's annual budget, small in isolation, yet multiplied across our 108-patient cohort, it equates to roughly 7.5 person-years of 1.5 °C-compatible carbon budget conserved each year at a single institution. Put another way, a single MRCP generates the same emissions as approximately 158 km of average passenger-vehicle driving, whereas a single EUS generates the equivalent of only 38 km.

Using the US Environmental Protection Agency Greenhouse Gas Equivalencies Calculator (17), the 1,552 kg CO_2_e saved across the 108 procedures is equivalent to 175 gallons of petrol consumed, 12,933 km of driving by an average passenger vehicle, the annual carbon dioxide absorption of 74 mature trees, or the electricity use of 1.6 average households for a year. On a per-patient basis, the 14.4 kg CO_2_e saved corresponds to roughly 120 km of driving or 6.2 L of petrol. These everyday comparisons translate an otherwise abstract carbon-accounting result into a tangible institutional and individual impact.

### Sensitivity analysis

EUS was the cleaner option in every scenario except the greenest grids (Table 5). The gap grew when the scanner was less busy: at our typical 22 scans per day, MRCP was 4.2 times higher than EUS, rising to roughly 8-fold at 10 scans and 16-fold at 5 scans, because the scanner's constant standby energy is then shared across fewer scans. Even at a very high 40 scans per day, MRCP was still approximately 2.6 times higher. Counting only the extra energy each scan adds, rather than the full standby load, narrowed the gap to 1.7-fold (MRCP 3.56 vs EUS 2.13 kg) but did not close it, so the advantage is not simply an artefact of how the fixed energy is allocated. Scan duration made little difference (4.2- to 4.8-fold from 20 to 40 minutes). The factor that really mattered was the grid: the gap fell to approximately 3-fold on a US grid and 2.6-fold on a UK grid, and the 2 became essentially equal below roughly 0.05 kg CO_2_e/kWh, where EUS's fixed water footprint sets a floor whereas MRCP's mostly-electric emissions keep falling.

**Table 5.** Sensitivity analysis of per-procedure carbon footprint across scanner utilization, energy-allocation method, and grid carbon intensity

Scenario	EUS (kg CO_2_e)	MRCP (kg CO_2_e)	Fold (MRCP/EUS)
Base case: ∼22 MRI/d, India grid (0.82), fully allocated	4.53	18.90	4.2×
MRI throughput 40/d (very high volume)	4.53	12.00	2.6×
MRI throughput 30/d	4.53	14.81	3.3×
MRI throughput 15/d	4.53	26.06	5.8×
MRI throughput 10/d	4.53	37.31	8.2×
MRI throughput 5/d (low volume)	4.53	71.06	15.7×
Marginal allocation (fixed standby and room excluded)	2.13	3.56	1.7×
US grid (0.37 kg CO_2_e/kWh)	2.55	8.53	3.3×
UK grid (0.207 kg CO_2_e/kWh)	1.84	4.77	2.6×
Low-carbon grid (0.05 kg CO_2_e/kWh)	1.15	1.15	1.0×
Hydro/nuclear-dominant grid (0.03 kg CO_2_e/kWh)	1.06	0.69	0.7×

MRI active-scan duration was additionally varied from 20 to 40 min at base utilization, changing the fold difference only modestly (4.2× to 4.8×). The water (Scope 3) component of EUS is grid-independent and was held constant in the grid analyses; because it forms a fixed floor for EUS, the intermodality advantage narrows and can reverse as grid carbon intensity approaches zero.

CO_2_e, carbon dioxide equivalent; EUS, endoscopic ultrasound; MRCP, magnetic resonance cholangiopancreatography.

## DISCUSSION

To our knowledge, this is among the first studies to compare the carbon footprint of EUS and MRCP at the patient level for intermediate-likelihood bile duct stones, and it demonstrates a clear and substantial difference: MRCP is 4.2 times more carbon-intensive than EUS. Across our 108-patient cohort, this translated into savings of more than 1.5 tons of CO_2_e, driven overwhelmingly by the MRI scanner rather than by any consumable or procedural factors.

A notable finding is that the MRI scanner's continuous standby power, drawn by the cryocooler to keep the superconducting magnet at its operating temperature, accounts for 23.4% of per-scan emissions during the dedicated standby state; because this 9-kW load also runs throughout active scanning and ready time, the cryocooler's continuous draw represents roughly 42% of per-scan MRCP emissions overall, a fixed energy cost with no counterpart in EUS. Our per-scan estimate of 23.04 kWh is consistent with independent measurements: Heye et al (11) reported a mean of 19.9 kWh per MRI examination using a comparable fully allocated method, and the 9 kW standby figure matches both the Philips manufacturer specification and measurements on similar 3 T systems (13,18).

In short, the EUS advantage depends on the setting. On a carbon-heavy grid like India's, EUS is approximately 4 times cleaner, and more so at the lower scan volumes typical of smaller centers. As the grid gets cleaner, the gap narrows, and below roughly 0.05 kg CO_2_e/kWh the 2 are effectively equal, because EUS's water use sets a floor whereas MRCP's largely electric footprint keeps falling. How the standby energy is counted matters too: the advantage is 4.2-fold when the always-on cryocooler load is shared across scans, and still 1.7-fold when only each scan's extra energy is counted. The case for EUS is therefore strongest where electricity is carbon-intensive and weakest, although not absent, where it is already clean.

The scope-wash step alone, using 75 L per cycle in a 4.5 kW machine, contributes more emissions than the endoscope itself. Hospitals aiming to reduce the environmental impact of EUS should therefore focus on low-water-consumption reprocessing equipment and energy-efficient washing machines rather than on the endoscopy procedure itself.

A practical difference between the modalities is timing: because EUS confirms stones in real time, it can be followed by same-session ERCP, whereas MRCP typically precedes ERCP by several days (a median of 8.5 vs 1 day in the FLUYT trial (19)). This interval allows spontaneous passage of small stones in a proportion of patients (20), which is 1 reason immediate ERCP is discouraged for small ductal stones (21). These are operational considerations rather than findings of the present study; our focus is the environmental comparison, and any such workflow advantages must be weighed against the invasiveness and sedation requirement of EUS relative to noninvasive MRCP.

From the patient's standpoint, MRCP also imposes considerable practical burdens, including confinement in a narrow bore for 25–30 minutes, exposure to loud gradient noise exceeding 100 dB, and claustrophobia that affects 4%–17% of patients referred for MRI. Patients with cardiac pacemakers, cochlear implants, certain aneurysm clips, or other ferromagnetic implants cannot undergo MRI at all, and severe obesity may rule out standard-bore scanners. EUS avoids these imaging-specific constraints and is generally well tolerated; it does, however, require deep sedation, with its attendant cardiorespiratory risk, and dedicated endoscopic expertise, considerations that do not apply to noninvasive MRCP.

A formal cost-effectiveness comparison between EUS and MRCP is difficult to generalize because costs and availability vary considerably across regions worldwide. EUS is an operator-dependent procedure that requires specialized endoscopic expertise, a defined learning curve, and is largely concentrated in tertiary and quaternary centers; patients often need to travel to access it. MRCP, by contrast, depends on the availability of an MRI scanner and radiology reporting expertise, and in many health systems, it is more widely accessible at peripheral and secondary centers. The relative price of each test, reimbursement structures, and the local concentration of skilled operators therefore vary widely between regions, and even between institutions within the same country.

For these reasons, a single cost-effectiveness verdict applicable everywhere is neither realistic nor useful. What our findings add, however, is a dimension that has so far been absent from such appraisals: the environmental cost. As health systems increasingly internalize the externalities of greenhouse gas emissions, the substantially lower carbon footprint of EUS becomes an additional factor to weigh alongside cost, availability, and local expertise when designing a diagnostic pathway for intermediate-likelihood choledocholithiasis.

Our findings align with the broader trend in gastroenterology sustainability research. The systematic review by Massironi et al (22) found MRI to be roughly 20-fold more carbon-intensive than ultrasound across 31 studies. Tan et al reported a reduction of 2,752 kg CO_2_e by replacing MR enterography with intestinal ultrasound in patients with inflammatory bowel disease (23). A consistent pattern is emerging: ultrasound-based modalities have a substantially smaller carbon footprint than MRI-based alternatives when the clinical indication is equivalent. Our study adds the first prospectively measured data point specifically for biliary disease. Reassuringly, our modeled MRCP figure aligns closely with direct measurement: in a prospective life-cycle assessment across 2 Australian hospitals, McAlister et al recorded a mean of 17.5 kg CO_2_e per MRI scan (18), close to our 18.90 kg CO_2_e for MRCP, with scanner standby energy the dominant contributor in both studies. That an independently and empirically measured MRI footprint falls so near our power-model estimate lends external support to the modeled MRCP value.

The emissions from any single procedure are small in absolute terms, but aggregated across institutional, national, and global practice, individually modest choices add up. Choosing EUS over MRCP when diagnostic accuracy is comparable saves 14.4 kg CO_2_e per patient, and the health harms of a warming climate fall hardest on the populations our hospitals serve.

Looking ahead, ESGE, British Society of Gastroenterology, and GI multisociety strategic plans have called for net-zero greenhouse gas emissions in GI endoscopy by 2050. The choice between EUS and MRCP for intermediate-likelihood CBD stones, where both are guideline-endorsed and diagnostically equivalent, presents a rare opportunity to exercise an environmental preference without any clinical trade-off. Carbon footprint warrants consideration alongside accuracy, safety, and cost-effectiveness in future clinical guideline frameworks.

This study has several strengths. It is among the first prospective, patient-level comparisons of the carbon footprints of EUS and MRCP, and it incorporates every major electricity component, including the continuous cryogenic standby power that most earlier radiology carbon assessments had not looked into. Procedure times were recorded individually rather than assumed, and the three-state MRI power model aligns closely with published measurements and manufacturer specifications, providing confidence in the estimates.

This study has some limitations. First, it is a single-center analysis; scan volumes, grid carbon intensity, and scanner models differ elsewhere, so absolute values are not directly generalizable. Second, MRCP emissions were derived from a validated engineering power model applied to real institutional scan volumes rather than measured for an individually enrolled MRCP cohort; consequently, this is a prospective EUS measurement benchmarked against a modeled MRCP comparator, not a randomized head-to-head trial, and per-patient MRCP scan durations were unavailable, so an average appointment profile was used (addressed by the duration sensitivity analysis). Third, allocation between EUS and MRCP followed physician discretion rather than randomization, although the per-procedure emission estimates are not sensitive to this. Fourth, a substantial share of the MRCP footprint derives from fixed cryocooler and room loads that persist regardless of individual scan volume; we therefore report both fully allocated and marginal estimates. Fifth, sedation materials were captured only as propofol vial glass, and the drug-substance manufacturing footprint and minor intravenous consumables were not separately modeled, although these are expected to be small. Sixth, patient travel was not captured; its inclusion would likely widen the gap in favor of EUS when same-session ERCP avoids a return visit. Finally, data were collected over a single calendar month, and results are presented as deterministic scenario estimates rather than probabilistic confidence intervals, reflecting that the principal inputs are fixed device power ratings; a year-long assessment would refine absolute figures but is unlikely to change the large difference between modalities.

MRCP generates 4.2 times the per-procedure carbon footprint of EUS for intermediate-likelihood choledocholithiasis, driven predominantly by the MRI scanner's 24/7 standby power, although this margin narrows on low-carbon electricity grids. Where both modalities are available, we recommend weighing carbon footprint alongside diagnostic accuracy, safety, and cost, particularly in carbon-intensive healthcare settings.

## CONFLICTS OF INTEREST

**Guarantor of the article:** Hardik Rughwani, DM.

**Specific author contributions:** H.R., R.K., N.J., D.N.R.: Concept and design. H.R., N.J., A.T., T.P., A.P., A.K.K.: Analysis and interpretation of data. D.M.d.J., M.R., S.L., D.N.R.: Critical revision of the article for important intellectual content. H.R., N.J., D.M.d.J., A.T., T.P., A.P., A.K.K., R.K., M.R., S.L., D.N.R.: Final approval of the article.

**Financial support:** None to report.

**Potential competing interests:** None to report.

**Study Highlights**

**WHAT IS KNOWN**

✓ Health care contributes nearly 5% of global greenhouse gas emissions, and medical imaging is highly energy-intensive.
✓ Endoscopic ultrasound (EUS) and magnetic resonance cholangiopancreatography (MRCP) have comparable diagnostic accuracy for intermediate-likelihood common bile duct stones.
✓ The environmental footprints of these 2 modalities had not been prospectively compared.

**WHAT IS NEW HERE**

✓ EUS generated 4.53 kg CO_2_e per procedure vs 18.90 kg for MRCP, making MRCP 4.2 times more carbon-intensive.
✓ The continuously running magnetic resonance imaging cryocooler was the largest fixed contributor to MRCP emissions (standby state alone: 23.4%), with no equivalent in EUS.
✓ Choosing EUS for 108 patients saved 1,552 kg CO_2_e, equal to the annual carbon uptake of 74 mature trees.
✓ When accuracy is comparable, EUS is the lower carbon choice, an advantage that is greatest in carbon-intensive electricity grids.

## ABBREVIATIONS:

AIG: Asian Institute of Gastroenterology
CBD: common bile duct
CO_2_e: carbon dioxide equivalent
ERCP: endoscopic retrograde cholangiopancreatography
ESGE: European Society of Gastrointestinal Endoscopy
EUS: endoscopic ultrasound
GHG: greenhouse gas
GI: gastrointestinal
IEC: Institutional Ethics Committee
MRCP: magnetic resonance cholangiopancreatography
MRI: magnetic resonance imaging
